# *In Vitro* and *In Vivo* Antidiabetic Effects of *Cratoxylum formosum* Leaf Extract in Low‐Dose Streptozotocin‐ and High‐Fat Diet‐Induced Diabetic Mice

**DOI:** 10.1155/jnme/8893700

**Published:** 2026-09-30

**Authors:** Nipaporn Muangchan, Suwattanee Kooptiwut, Anchalee Vattarakorn, Bancha Yingngam, Kanchana Suksri

**Affiliations:** ^1^ Department of Biopharmacy Faculty of Pharmaceutical Sciences Ubon Ratchathani University Ubon Ratchathani Thailand ubu.ac.th; ^2^ Department of Physiology Faculty of Medicine Siriraj Hospital Mahidol University Bangkok Thailand mahidol.ac.th; ^3^ Department of Pharmaceutical Chemistry and Technology Faculty of Pharmaceutical Sciences Ubon Ratchathani University Ubon Ratchathani Thailand ubu.ac.th; ^4^ Division of Pharmacology and Biopharmaceutical Sciences Faculty of Pharmaceutical Sciences Burapha University Chonburi Thailand buu.ac.th

**Keywords:** α-amylase, α-glucosidase, *Cratoxylum formosum*, diabetes, insulin, neuropeptide Y, phenolic compound

## Abstract

*Cratoxylum formosum* (CF) is a phenolic‐rich plant and exhibits *in vitro* antioxidant properties, yet its in vivo therapeutic potential remains uncharacterized. This study evaluated the antidiabetic efficacy of an ethanolic CF leaf extract using high‐fat diet/streptozotocin‐induced diabetic mice, complemented by enzymatic and INS‐1 rat insulinoma cell line assays. This study found that 5‐O‐caffeoylquinic acid (5‐CQA) was identified as the major phenolic compound in the CF leaf extract. In vitro, CF extract inhibited α‐amylase and α‐glucosidase activities and protected INS‐1 cells from high‐glucose‐induced cytotoxicity. In diabetic mice, CF extract administration attenuated hyperglycemia, partially preserved serum insulin and C‐peptide levels, and suppressed serum neuropeptide Y (NPY) concentrations. Histological analysis confirmed the preservation of pancreatic islet architecture and pancreatic β‐cell mass. These findings indicate that CF extract supports glycemic regulation and pancreatic β‐cell preservation, warranting further exploration as a candidate for diabetes management.

**Highlights**
• 5‐O‐caffeoylquinic acid (5‐CQA) is the predominant phenolic antioxidant in *Cratoxylum formosum* (CF) extract.
• CF extract inhibits α‐amylase and α‐glucosidase activities *in vitro.*
• CF attenuates hyperglycemia, restores insulin/C‐peptide levels, and suppresses serum NPY in diabetic mice.
• CF preserves pancreatic β‐cell mass *in vivo* and enhances INS‐1 cell viability under high‐glucose conditions.

## 1. Introduction

Type 2 diabetes is a multifactorial disease commonly associated with insulin resistance, impaired insulin secretion, hyperglycemia, and disrupted metabolic function and is often exacerbated by obesity and unhealthy dietary patterns [1]. In recent years, the focus of alternative diabetes therapies has shifted toward polyphenols derived from edible plants and their antioxidant properties, which may help mitigate the pathophysiology of type 2 diabetes [2]. Several studies have suggested that consuming polyphenol‐rich foods can protect pancreatic β cells from oxidative damage [3, 4]. Additionally, various polyphenol‐rich plants exhibit antidiabetic properties by either enhancing antioxidant or anti‐inflammatory defenses [5] or inhibiting the activities of carbohydrate‐hydrolyzing enzymes, such as α‐glucosidase and α‐amylase [6]. For instance, gallic acid‐a phenolic compound‐has been reported to increase insulin secretion and support the survival of pancreatic β cells under high‐glucose conditions [7].

*Cratoxylum formosum* (CF, Jack) Benth. & Hook.f. ex Dyer, a plant native to Southeast Asia, is rich in phenolic compounds and has displayed promising therapeutic effects under various health conditions. Its documented properties include anti‐inflammatory, antioxidant, antimicrobial, anticancer, and wound‐healing activities [8]. The identification of polyphenols in CF extract has been reported by Yingngam et al., who reported that 5‐*O*‐caffeoylquinic acid (5‐CQA), a class of chlorogenic acid, was the major phenolic compound in the CF extract [8, 9]. Neuropeptide Y (NPY) is a key orexigenic peptide secreted in the arcuate nucleus of the hypothalamus. It promotes food intake and reduces energy expenditure [10]. In a type 2 diabetes mellitus rat model, a time‐dependent increase in hypothalamic NPY levels and mRNA expression was detected. [11]. Chlorogenic acid has been shown to reduce hyperphagia, weight gain, and fat accumulation in Wistar rats by downregulating the expression of the appetite‐regulating hormone NPY‐an, thus reducing orexigenic signaling [12]. Moreover, CF has been shown to inhibit α‐glucosidase and α‐amylase activities *in vitro* [13]. To date, no studies have systematically investigated the effects of CF extract on diabetic animal models or pancreatic β‐cell lines or on NPY modulation. Thus, this study aimed to evaluate both the *in vivo* and *in vitro* effects of CF extract on glucose metabolism and β‐cell protection. The *in vivo* model employed a combination of low‐dose streptozotocin (STZ) injection and a high‐fat diet (HFD) to induce type 2 diabetes in a mouse model, whereas the *in vitro* investigations were conducted using a rat insulinoma cell line (INS‐1 cells) to assess direct cellular effects.

## 2. Materials and Methods

### 2.1. Preparation of the CF Extract

Young CF leaves were harvested in Ubon Ratchathani Province, Thailand. The plant material was authenticated by Assoc. Prof. Bancha Yingngam, Department of Pharmaceutical Chemistry and Technology, Faculty of Pharmaceutical Sciences, Ubon Ratchathani University, who also performed the extraction together with the research team. Fresh leaves were oven‐dried in a hot‐air oven at 50°C for 48 h until a constant weight was obtained. The dried leaves were ground into powder and stored in sealed plastic bags protected from light until extraction. The extraction procedure was adapted from the optimized phenolic extraction method previously reported for *C. formosum* leaves [8]. Briefly, the dried leaf powder was mixed with aqueous ethanol (50% ethanol‐water, v/v) at a solid‐to‐solvent ratio of 1:30 g/mL and extracted at 65°C for 15 min in a single extraction cycle. The mixture was allowed to cool to room temperature and then filtered. The ethanolic solvent was removed under reduced pressure at no more than 40°C, and the concentrated extract was dried to obtain the CF leaf extract. The dried extract was kept at −20°C until further use.

### 2.2. Determination of Total Phenolic Content (TPC)

The TPC of the CF leaf extract was quantified by the Folin–Ciocalteu colorimetric assay following the procedure reported by Singleton et al. [14], with minor modifications. In brief, 25 μL of 0.2 N Folin–Ciocalteu reagent was mixed with 25 μL of CF leaf extract at doses varying from 0.0156 to 1 mg/mL. After half an hour, the reaction mixture was left in the dark at room temperature. After 100 μL of 7% (w/v) sodium carbonate solution was added, the mixture was incubated for another 5 min under the same conditions. The absorbance was recorded at 730 nm using a microplate reader (Omega, Germany). The TPC was reported as milligrams of gallic acid equivalents per gram of dried extract (mg GAE/g dry weight) and was calculated from a gallic acid calibration curve. All measurements were performed in triplicate.

### 2.3. Examination of Antioxidant Activity Via a DPPH^•^ Radical Scavenging Assay

The free radical scavenging capacity of the CF extract against 2,2‐diphenyl‐1‐picrylhydrazyl (DPPH^•^) was evaluated using a modified protocol based on Clarke [15]. Briefly, 50 μL of CF extract prepared in 5% DMSO at dosages varying from 0.0156 to 1 mg/mL was added to 150 μL of 0.3 M DPPH solution in absolute ethanol in a 96‐well microplate. The mixture was gently mixed for 2 min and then incubated at room temperature in the dark for 30 min. The absorbance was recorded at 517 nm using a spectrophotometer (Omega, Germany). Gallic acid was used as the reference antioxidant. The DPPH^•^ radical scavenging activity was expressed as a percentage and calculated using the following equation:

(1) DPPH scavenging activity %=Control absorbance−Sample absorbanceControl absorbance×100.

### 2.4. Examination of Antioxidant Activity via the ABTS^•+^ Radical Scavenging Assay

The ABTS assay was performed according to a modified procedure adapted from Ozgen et al. [16]. To create ABTS^•+^ radical cations, 7 mM ABTS was combined with 2.45 mM potassium persulfate in distilled water. The mixture was then incubated at room temperature for 12–16 h. The resulting solution was diluted with methanol to produce an absorbance of 0.7 ± 0.02 at 734 nm. The leaf extract (at concentrations of 10, 50, 100, and 200 μg/mL) was mixed with 100 μL of the ABTS^•+^ working solution and allowed to react at room temperature for 6 min. The absorbance was measured at 734 nm using a microplate reader (Omega, Germany). The ABTS^•+^ radical scavenging activity was calculated as a percentage and computed using the equation below.

(2) ABTS scavenging activity %=Control absorbance−Sample absorbanceControl absorbance×100.

### 2.5. HPLC Determination of 5‐O‐Caffeoylquinic Acid in the CF Extract

The chromatographic profile of the CF leaf extract batch used in this study was determined using high‐performance liquid chromatography coupled with a diode‐array detector (HPLC‐DAD; Dionex Ultimate 3000 UHPLC, Thermo Fisher Scientific, USA). The dried extract was dissolved in methanol at 10 mg/mL, sonicated for 10 min, and filtered through a 0.45‐μm membrane filter. Separation was performed using a Hypersil GOLD C18 column (250 × 4.6 mm, 5‐μm particle size) maintained at 35 ± 1°C. The mobile phase consisted of 50 mmol/L phosphoric acid in water (Solvent A) and acetonitrile (Solvent B). The gradient program was as follows: 5% B for 0–5 min, a linear increase to 50% B from 5–50 min, 50% B from 50–60 min, and a return to 5% B within 2 min. The flow rate was 1 mL/min, the injection volume was 10 μL, and detection was performed at 325 nm. An authentic 5‐CQA standard was used for identification and quantification. Compound identity was confirmed by comparison of the retention time and UV absorption spectrum and by sample spiking. Quantification was performed using an external‐standard calibration curve over the range of 1–100 μg/mL, with a coefficient of determination (*R* ^2^) of 0.9993. The results are expressed as milligrams of 5‐CQA per gram of dried extract. All measurements were performed in triplicate.

### 2.6. Determination of the Inhibitory Effects of the CF Extract on α‐Amylase and α‐Glucosidase Activities

The α‐amylase and α‐glucosidase inhibitory activities of CF extract (0.125–2 mg/mL) were evaluated using colorimetric kits (ab283391 and ab284520; Abcam, UK) with minor modifications [17]. Briefly, 50 mL of enzyme solution was preincubated with 50 mL of extract or acarbose (positive control) at 37°C for 10 min. The reaction was initiated by adding 50 mL of substrate (starch for α‐amylase; *p*‐nitrophenyl, and‐α‐D‐glucopyranoside for α‐glucosidase) and monitored at 405 nm using a microplate reader (Omega, Germany). The reaction was monitored by measuring the absorbance at 405 nm using a microplate reader (Omega, Germany) under kinetic conditions for 5–20 min at room temperature. Two time points (t1 and t2) within the linear phase of the reaction curve were selected, and the corresponding absorbance values (OD1 and OD2) were used to calculate the reaction rate and percentage inhibition. The IC50 values were estimated from concentration–response curves.

### 2.7. Direct Effect of CF Extract in INS‐1 Rat Insulinoma β Cell Under High Glucose Condition

The direct effect of CF extract on protection of pancreatic β cells under high glucose exposure was evaluated in INS‐1 rat insulinoma β cells using the MTT assay [18]. Cells (10,000 cells/well) were seeded in 96‐well plates. Cytotoxicity was assessed by treating cells with CF extract (1–500 mg/mL) in RPMI‐1640 medium for 48 h. Cytoprotection was evaluated by exposing cells to normal (11.1 mM) or high (40 mM) glucose with or without CF extract for 72 h. Following treatment, cells were incubated with MTT solution for 4 h (37°C, 5% CO2). Formazan crystals were dissolved in DMSO, and absorbance was measured at 570 nm using a microplate reader (Omega, Germany). Cell viability was expressed as a percentage relative to the untreated control using the following equation:

(3) Cell viability %=AbsorbanceSampleAbsorbanceControl×100.

### 2.8. Animal Study

ICR male mice were purchased from the National Laboratory Animal Center at Mahidol University in Thailand. The animals were housed in a controlled setting with a 12 h light/dark cycle and unrestricted access to normal food and potable water. The animal experiments followed the National Institutes of Health guidelines (8th edition) and were approved by the Institutional Animal Care and Use Committee (IACUC), Faculty of Medicine, Siriraj Hospital, Mahidol University (Certificate of Approval No. 017/2023).

### 2.9. Experimental Design

The experimental design for the animals is depicted in Figure 1. Fifty‐six 6‐week‐old male ICR mice were initially allocated to seven groups (*n* = 8/group): normal control, normal mice treated with 500 mg/kg b.w. CF extract, untreated diabetic mice (DM), DM + 50 mg/kg b.w. CF extract, DM + 100 mg/kg b.w. CF extract, DM + 500 mg/kg b.w. CF extract, and DM + 100 mg/kg b.w. acarbose. The initial sample size was selected on the basis of power considerations from previous STZ‐induced diabetic mouse studies [19] and to allow for potential attrition after diabetes induction. Diabetes was induced by HFD feeding combined with intraperitoneal injection of low‐dose STZ (35 mg/kg b.w.). Hyperglycemic mice were then randomly assigned to the treatment groups using simple randomization. The CF extract doses (50, 100, and 500 mg/kg b.w.) were selected to cover low, intermediate, and high oral dose levels and were guided by prior *in vivo* use of CF extract in rodents [20]. Acarbose (100 mg/kg b.w.) was selected as a positive control because it is a standard intestinal alpha‐glucosidase inhibitor and has been used as a comparator in HFD/STZ‐induced diabetic mouse studies and for OGTT‐based antidiabetic evaluation [21]. All treatments were administered once daily by oral gavage for 4 weeks. Body weight (B.W.), food intake, and fasting blood sugar (FBS) levels were monitored weekly. At Week 4 of treatment, an oral glucose tolerance test (OGTT) was performed. The mice were then euthanized, and blood and organs were collected for biochemical and histological analysis. Although blinding was not feasible during daily oral administration because the extract and control solutions differed visibly, potential bias was minimized by random allocation, standardized handling and gavage procedures, objective endpoint measurement, and blinding of the investigators during ELISA, histological image acquisition, and morphometric analysis. The final sample sizes were *n* = 6–8/group for longitudinal glycemic and body/organ weight outcomes, *n* = 4/group for serum ELISA measurements, and *n* = 4–5/group for histological assessment, as indicated in the figure legends.

**FIGURE 1:**
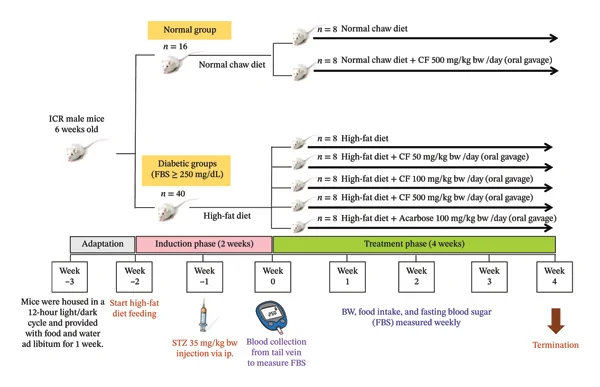
Experimental design for the animals.

### 2.10. OGTT

The effect of CF extract on reducing postprandial glucose levels was evaluated via the OGTT [21]. This assay was conducted on the 29th day of treatment. The mice were fasted for 8–12 h before the test. An oral glucose load (2000 mg glucose/kg b.w.) was administered 30 min after each treatment, and blood glucose was measured before (baseline) and after (30, 60, and 90 min) glucose loading using a glucometer strip test (Accu‐Chek).

### 2.11. Measurements of Serum Insulin, C‐Peptide, and NPY Levels

At the end of the 4th week, the mice were euthanized by intraperitoneal injection of pentobarbital sodium (120 mg/kg b.w.). Blood samples were collected, and serum was separated and stored at −80°C until analysis. Serum insulin, C‐peptide, and NPY levels were determined using commercial ELISA kits according to the manufacturers’ protocols. Insulin levels were measured using a Mouse INS (Insulin) ELISA Kit (E‐EL‐M3119; Elabscience Biotechnology Co., Ltd., Wuhan, China; detection range 78.13–5000 pg/mL; sensitivity 46.88 pg/mL). C‐peptide levels were measured using a Mouse C‐P (C‐Peptide) ELISA Kit (E‐EL‐M0354; Elabscience; detection range 0.16–10 ng/mL; sensitivity 0.09 ng/mL). NPY was measured using a Mouse NPY ELISA Kit (E‐EL‐M0820; Elabscience; detection range 31.25–2000 pg/mL; sensitivity 18.75 pg/mL). The absorbance was measured at 450 nm using a microplate reader, and concentrations were calculated from standard curves [22–24].

### 2.12. Histological Analysis of the Pancreas

The pancreatic tissues were fixed in 10% neutral buffered formalin for 2 weeks, embedded in paraffin, and sectioned at 3 μm thickness. Sections were stained with hematoxylin and eosin (H&E) for assessment of pancreatic islet morphology. For immunohistochemistry, the sections were stained with an anti‐insulin antibody to identify pancreatic β cells. Digital images were captured under a light microscope, and morphometric analysis was performed using image‐analysis software by investigators who were blinded to treatment allocation. The islet area and insulin‐positive β‐cell area were quantified following established histomorphometric principles for assessing pancreatic β‐cell mass [25]. The percentages of islet and pancreatic β‐cell areas were calculated using the following equations:

(4) Islet area %=Islet areaTotal area×100,

(5) Pancreatic β cell area %=Isulin positive areaTotal section area×100.

### 2.13. Statistical Analysis

All the quantitative parameters, including body weight, organ weight, blood glucose concentration, serum insulin concentration, C‐peptide level, NPY level, OGTT data, and pancreatic islet and β‐cell areas, are summarized as the mean ± standard error of the mean (SEM). Before the data were subjected to parametric analysis, the data distribution and variance assumptions were assessed using the Shapiro–Wilk test for normality and Levene’s or Brown–Forsythe test for homogeneity of variance, as appropriate. For single time‐point comparisons among groups, one‐way ANOVA followed by Tukey’s post hoc multiple‐comparison test was used when the assumptions were satisfied. When the assumptions were not satisfied, the corresponding nonparametric alternative was considered. Longitudinal FBS and OGTT glucose curves were analyzed using two‐way repeated‐measures ANOVA, with treatment group as the between‐subject factor and time as the within‐subject repeated factor, followed by multiple‐comparison testing. In addition, the OGTT area under the curve (AUC) was calculated using the trapezoidal method and compared among groups using one‐way ANOVA with Tukey’s post hoc test. A *p* value less than 0.05 was considered to indicate statistical significance.

## 3. Results and Discussion

### 3.1. TPC of the CF Leaf Extract

The Folin–Ciocalteu colorimetric assay was employed to quantify the TPC of the CF extract. As presented in Table 1, the TPC of the CF extract was 111.11 ± 3.03 mg GAE/g extract. The analysis was performed in triplicate to ensure the reliability and reproducibility of the results.

**TABLE 1:** Total phenolic content (TPC) and free radical scavenging activity of the CF extract.

	TPC (mg GAE/g extract)	Antioxidant activity (IC_50_)	
		DPPH^•^ assay	ABTS^•+^ assay
CF extract	111.11 ± 3.03	94.91 ± 9.65 μg/mL	78.13 ± 2.38 μg/mL
Gallic acid		7.88 ± 0.36 μg/mL	7.31 ± 0.32 μg/mL

*Note:* TPC and antioxidant activity of the CF extract. TPC and antioxidant activity were compared to those of a phenolic compound (gallic acid). Antioxidant activities against DPPH^•^ and ABTS^•+^ free radicals with IC_50_ values are presented as the means ± SEMs (*N* = 3).

### 3.2. Radical Scavenging Activity of the CF Extract

The antioxidant activity of the CF leaf extract was assessed using DPPH^•^ and ABTS^•+^ radical scavenging assays. As shown in Table 1, the CF extract demonstrated notable antioxidant potential, with IC_50_ values of 94.91 ± 9.65 μg/mL in the DPPH^•^ assay and 78.13 ± 2.38 μg/mL in the ABTS assay. In comparison, the standard antioxidant compound gallic acid exhibited significantly lower IC_50_ values (7.88 ± 0.36 μg/mL and 7.31 ± 0.32 μg/mL) in the DPPH^•^ and ABTS^•+^ assays, respectively, suggesting that the free radical scavenging capacity of the CF extract is lower than that of gallic acid. Further investigations are warranted to identify the specific bioactive constituents responsible for the antioxidant activity of the CF extract.

### 3.3. HPLC Characterization of the CF Leaf Extract

The HPLC‒DAD chromatogram of the CF extract recorded at 325 nm showed several detectable peaks, including a prominent peak at a retention time of 15.40 min. This peak was identified as 5‐CQA by comparison of its retention time and UV absorption spectrum with those of the authentic standard and by sample spiking (Figure 2). The calibration curve showed good linearity over the tested concentration range (*R* ^2^ = 0.9993). The 5‐CQA content of the extract was 243.74 ± 0.93 mg/g dry extract. Other chromatographic peaks were not structurally identified and were therefore not assigned to specific compounds.

**FIGURE 2:**
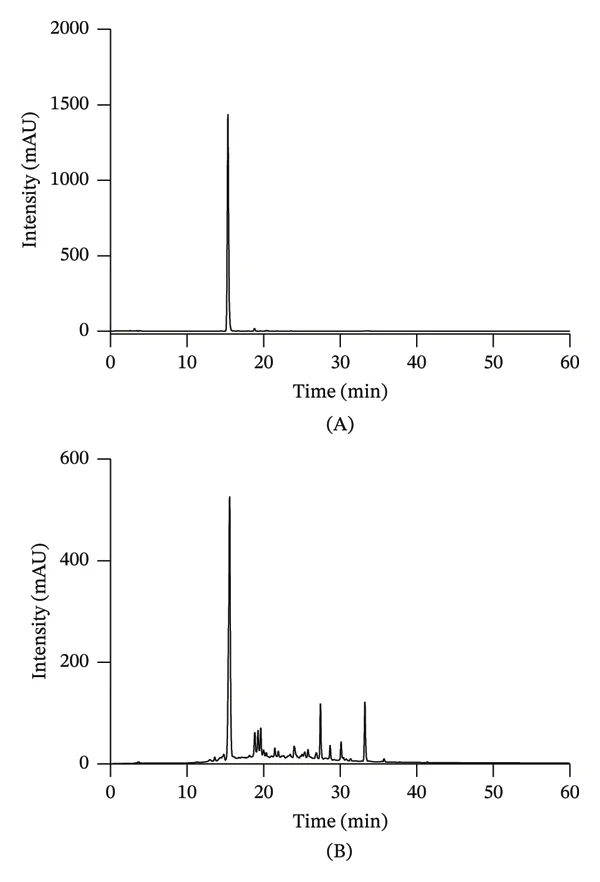
HPLC‐DAD chromatograms recorded at 325 nm: (A) authentic 5‐O‐caffeoylquinic acid (5‐CQA) standard and (B) *Cratoxylum formosum* leaf extract. The 5‐CQA peak in the extract was identified by comparison of its retention time and UV absorption spectrum with those of the authentic standard and confirmed by sample spiking.

### 3.4. *In vitro* Inhibitory Effects of CF Leaf Extract on α‐Amylase and α‐Glucosidase Activity

The *in vitro* inhibitory activities of the CF leaf extract on the carbohydrate‐digesting enzymes α‐amylase and α‐glucosidase were assessed using a colorimetric enzyme activity assay. As shown in Table 2, the CF leaf extract inhibited both enzymes, with IC_50_ values of 1.14 ± 0.38 mg/mL for α‐amylase and 1.83 ± 0.42 mg/mL for α‐glucosidase. These results indicate that the extract has a stronger inhibitory effect on α‐amylase than on α‐glucosidase and that the CF leaf extract may have the potential to modulate postprandial glucose levels by interfering with carbohydrate digestion. Further studies are warranted to elucidate the active constituents responsible for these inhibitory effects and explore their potential therapeutic applications in managing hyperglycemia and diabetes.

**TABLE 2:** Effects of CF extract on α‐amylase and α‐glucosidase activities.

	α‐Amylase inhibition (IC_50_)	α‐Glucosidase inhibition (IC_50_)
CF extract	1.14 ± 0.38 mg/mL	1.83 ± 0.42 mg/mL
Acarbose	2.67 × 10^−4^ ± 4 × 10^−6^ mg/mL	0.92 ± 0.19 mg/mL

*Note:* The *in vitro* activity of the CF extract against α‐amylase and α‐glucosidase activities was measured using a colorimetric enzyme activity assay. This activity was compared to that of a positive control inhibitor (acarbose). The inhibitory activities (IC_50_) of the CF extract on α‐amylase and α‐glucosidase activities are expressed as the means ± SEMs (*N* = 3).

### 3.5. *In vivo* Inhibitory Activities of CF Leaf Extract in Diabetic Mice

The antidiabetic effects of CF leaf extract were further evaluated in a type 2 diabetic mouse model. Diabetic mice were treated with CF extract at doses of 50, 100, and 500 mg/kg b.w., as well as with the standard antidiabetic agent acarbose, for 4 weeks. FBS levels and B.W. were monitored weekly throughout the study. As shown in Figure 3, the FBS levels in all treatment groups were assessed over the 4 weeks. Compared with the untreated diabetic group, the CF extract group exhibited significant antihyperglycemic effects, leading to reductions in blood glucose levels of 15.27%, 20.47%, and 29.31% at doses of 50, 100, and 500 mg/kg b.w., respectively, as presented in Table 3. These findings suggest that CF extract has a dose‐dependent glucose‐lowering effect on diabetic mice, with the highest dose (500 mg/kg b.w.) resulting in the most pronounced reduction in FBS levels.

**FIGURE 3:**
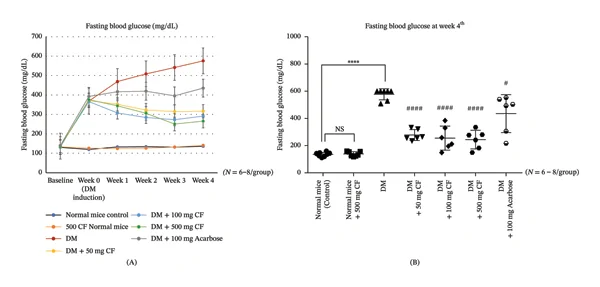
Effects of CF extract on the FBS level in all groups over 4 weeks. (A) Fasting blood sugar (FBS) levels were monitored weekly for 4 weeks of treatment. The data are expressed as the mean ± SEM (*n* = 6–8/group). Longitudinal FBS profiles were analyzed using two‐way repeated‐measures ANOVA. (B) Fasting blood sugar levels at Week 4 are presented as the mean ± SEM and were analyzed by one‐way ANOVA with Tukey’s multiple‐comparison test (^∗∗∗∗^ *p* < 0.0001 vs. normal control mice; ^#^ *p* < 0.05 vs. DM; ^####^ *p* < 0.0001 vs. DM).

**TABLE 3:** Effects of CF extract on fasting blood glucose levels in diabetic mice.

Groups	Fasting blood sugar (mg/dL)		% glucose reduction
	Week 0 (DM induction)	Week 4	
Normal mice control	119.75 ± 3.35	135.88 ± 9.07	−13.47
500 mg CF normal mice	125.92 ± 6.72	140.79 ± 3.95	−11.81
DM	368.54 ± 24.39	575.67 ± 44.56^∗∗∗∗^	−56.20
DM + 50 mg/kg b.w. CF	371.21 ± 55.11	314.54 ± 98.08^####^	15.27
DM + 100 mg/kg b.w. CF	366.04 ± 55.86	291.13 ± 100.16^####^	20.47
DM + 500 mg/kg b.w. CF	375.96 ± 46.06	265.75 ± 94.01^####^	29.31
DM + 100 mg/kg b.w. acarbose	392.83 ± 52.78	435.33 ± 150.66^#^	−10.82

*Note:* FBS levels at Weeks 0 and 4. The data are expressed as the mean ± SEM (*n* = 6–8/group). Significant differences between groups at Week 4 were determined using one‐way ANOVA with Tukey’s multiple‐comparison test (^∗∗∗∗^ *p* < 0.0001 vs. normal control mice;^#^ *p* < 0.05 vs. DM; ^####^ *p* < 0.0001 vs. DM). % glucose reduction = [(FBS at Week 0 ‐FBS at Week 4)/FBS at Week 0] × 100.

### 3.6. Effects of CF Extract on OGTT Results

To further investigate the antidiabetic potential of CF extract, its effect on postprandial glucose levels was assessed using an OGTT. As shown in Figure 4, compared with no treatment, treatment with CF extract improved glucose tolerance in diabetic mice. Repeated‐measures analysis was used for the glucose–time curves, while AUC values were calculated to quantify the cumulative postchallenge glycemic response. Although CF extract did not fully restore glucose tolerance to normal control levels, AUC analysis indicated a marked improvement in postprandial glucose handling, particularly at higher CF doses.

**FIGURE 4:**
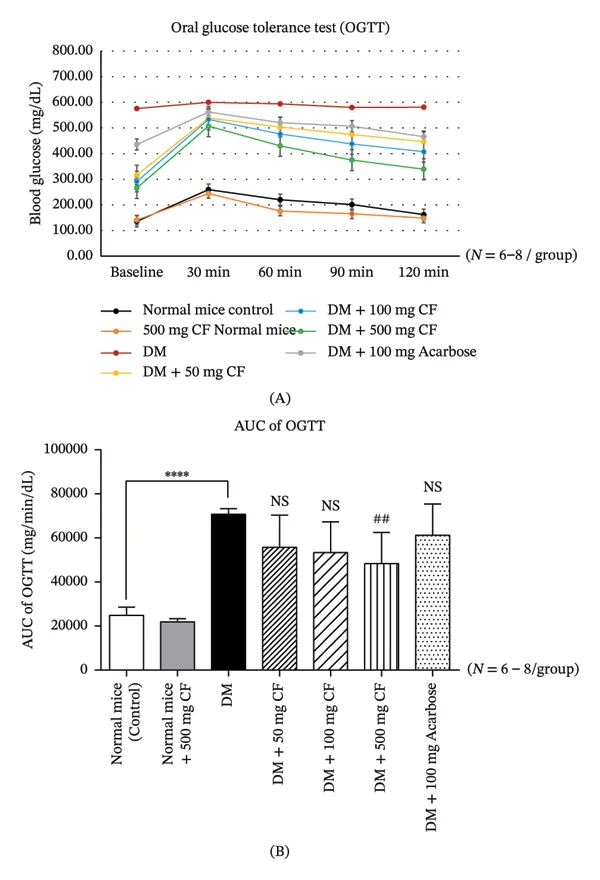
Effects of CF extract on postprandial glucose levels. (A) Blood glucose levels determined using the OGTT. The data are expressed as the mean ± SEM (*n* = 6–8/group), and the time course profile was analyzed by two‐way repeated‐measures ANOVA with treatment group and time as factors. (B) AUC of postprandial glucose levels calculated using the trapezoidal method and presented as the mean ± SEM. AUC values were compared using one‐way ANOVA with Tukey’s multiple‐comparison test (^∗∗∗∗^ *p* < 0.0001 vs. DM; ^#^ *p* < 0.05 vs. DM; ^##^ *p* < 0.01 vs. DM).

### 3.7. Effects of CF Extract on Maintaining Body Weight, Visceral Fat, Skeletal Muscle, and Pancreas Weights

The effects of CF extract on B.W., pancreatic weight, and insulin‐targeting organs, including visceral fat and skeletal muscle, were also evaluated. As shown in Figure 5, diabetes adversely affected these parameters, as observed in the untreated diabetic (DM) group. Treatment with CF extract led to a dose‐dependent improvement in pancreatic weight, with the highest dose (500 mg/kg b.w. CF) restoring pancreatic weight close to normal (0.39 g). These findings suggest that CF extract may have protective or restorative effects on pancreatic tissue, potentially by supporting pancreatic β‐cell function. Interestingly, the CF extract also influenced visceral fat accumulation in diabetic mice. The mice treated with 100 mg/kg b.w. of CF presented the highest visceral fat mass (0.43 g), whereas the 500 mg/kg b.w. CF group presented a slightly lower fat mass (0.38 g). This pattern suggests a complex dose‐response relationship, indicating that while CF extract may promote fat accumulation at moderate doses, higher doses may have different metabolic effects on adipose tissue regulation. Compared with normal mice (1.41 g), untreated diabetic mice (0.62 g) exhibited significant muscle wasting, a common consequence of diabetes‐related catabolic processes. CF extract treatment mitigated this muscle loss, with the highest dose (500 mg/kg b.w. CF) restoring skeletal muscle weight to 1.25 g, which is near normal. Notably, the CF extract at all the tested doses can preserve muscle mass in diabetic mice. These findings highlight the potential of CF extract in mitigating the adverse effects of diabetes on metabolic organs.

**FIGURE 5:**
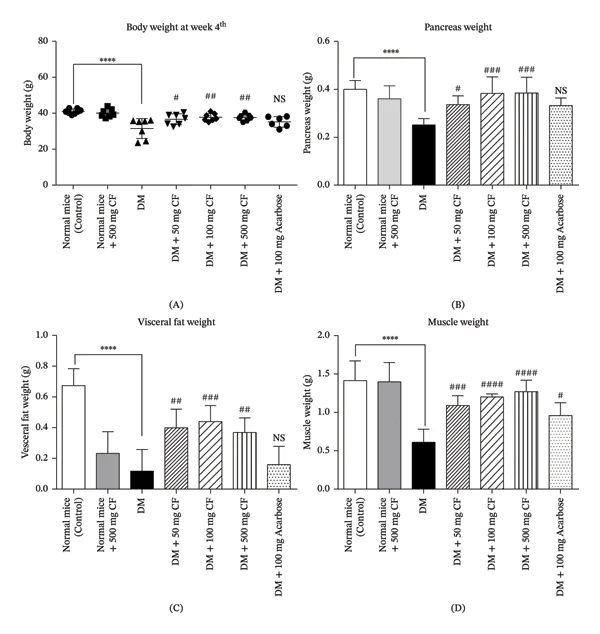
Effects of CF extract on body weight and organ weight. Effects of CF extract on body weight (A) and the weights of the pancreas (B), visceral fat (perigonadal) (C), and skeletal muscle (quadriceps + hamstrings + gastrocnemius) (D) at Week 4 (*n* = 6–8/group). The data are expressed as the mean ± SEM. Significant differences between groups were analyzed by one‐way ANOVA with Tukey’s multiple‐comparison test (^∗∗∗∗^ *p* < 0.0001 vs. normal control; ^#^ *p* < 0.05 vs. DM; ^##^ *p* < 0.01 vs. DM; ^###^ *p* < 0.001 vs. DM; ^####^ *p* < 0.0001 vs. DM).

### 3.8. Effects of CF Extract on Serum Insulin, C‐Peptide, and NPY Levels

To evaluate the effect of CF extract on pancreatic β‐cell function, serum insulin and C‐peptide levels were measured using an enzyme‐linked immunosorbent assay (ELISA). As shown in Figure 6, compared with normal diabetic mice, untreated diabetic mice exhibited significantly lower serum insulin and C‐peptide levels, indicating pancreatic dysfunction. The administration of CF extract resulted in dose‐dependent increases in insulin and C‐peptide levels in diabetic mice. Additionally, the effects of CF extract on serum levels of NPY, a key hormone involved in appetite regulation, were investigated. Diabetic mice exhibited markedly elevated NPY levels, which is consistent with the dysregulation of appetite control associated with diabetes. Notably, the highest dose of CF extract (500 mg/kg b.w.) significantly reduced NPY levels, which were closer to those of normal control mice.

**FIGURE 6:**
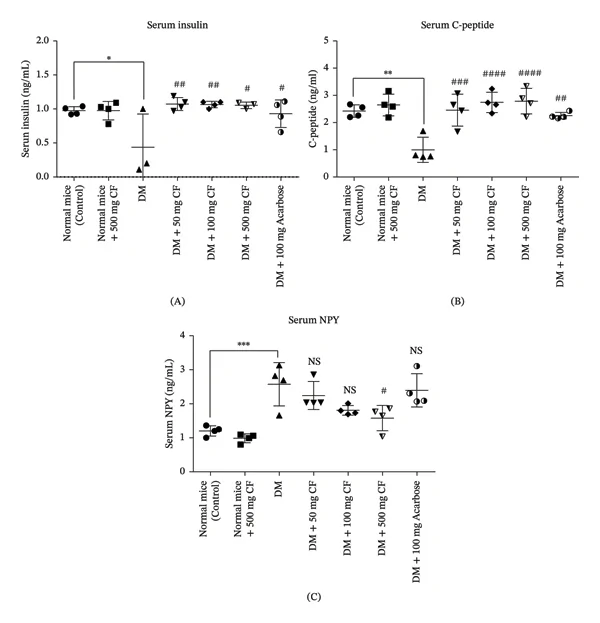
Effects of CF extract on serum insulin, C‐peptide, and NPY levels. Serum insulin (A), C‐peptide (B), and neuropeptide Y (NPY) (C) levels were measured at Week 4. The data are presented as the mean ± SEM (*n* = 4/group). Significant differences between groups were determined by one‐way ANOVA with Tukey’s multiple‐comparison test (^∗∗^ *p* < 0.01 vs. normal control; ^##^ *p* < 0.01 vs. DM; ^###^ *p* < 0.001 vs. DM; ^####^ *p* < 0.0001 vs. DM). Effects of CF extract on preserving pancreatic β‐cell mass.

To investigate the effects of CF extract on the preservation of pancreatic β‐cell mass, the islet and pancreatic β‐cell areas were measured through histological analysis. The results presented in Figure 7 correlate with the observed serum insulin and C‐peptide levels, providing further insights into the impact of the extract on pancreatic function. Treatment with CF extract led to a dose‐dependent improvement in both islet and pancreatic β‐cell areas. Specifically, compared with the control treatment, the 500 mg/kg b.w. CF extract treatment resulted in the most significant preservation of pancreatic islet and β‐cell areas, suggesting that the CF extract may have a protective effect on pancreatic β‐cells.

**FIGURE 7:**
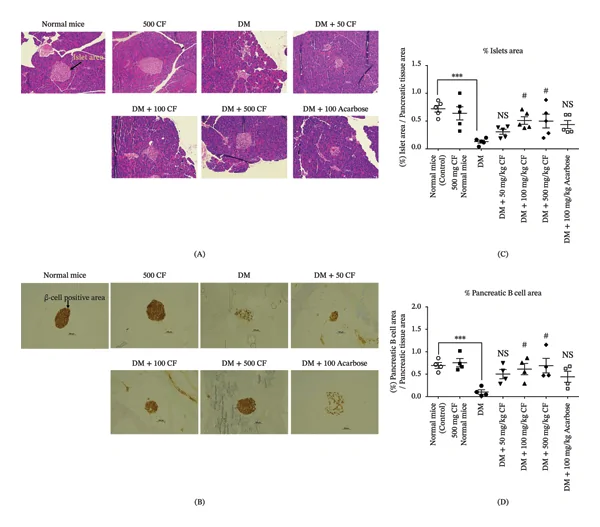
Effects of CF extract on pancreatic islets and β‐cell areas. The area and morphology of pancreatic islets were evaluated using H&E staining (A). The area and morphology of pancreatic β‐cells were determined by immunohistochemical staining with an insulin antibody (B). The % islet area (C) and % pancreatic β‐cell area (D) are presented as the mean ± SEM (*n* = 4–5/group). Significant differences between groups were determined by one‐way ANOVA with Tukey’s multiple‐comparison test (^∗∗∗^, *p* < 0.001 vs. normal control; ^#^, *p* < 0.05 vs. DM).

### 3.9. Direct Effect of CF Extract in INS‐1 Cells Under High Glucose Condition

To evaluate the direct effects of CF leaf extract on protection of pancreatic β‐cell damage under high glucose conditions, INS‐1 cells were treated and assessed using the MTT assay. As shown in Figure 8A, the CF extract did not induce cytotoxicity in INS‐1 cells at concentrations ranging from 1–500 μg/mL. Notably, compared with the normal‐glucose control treatment, treatment with the CF extract at 5 and 10 μg/mL significantly increased the viability of INS‐1 cells under high‐glucose conditions (Figure 8B), suggesting the potential protective trends against pancreatic β‐cell damage under hyperglycemic conditions.

**FIGURE 8:**
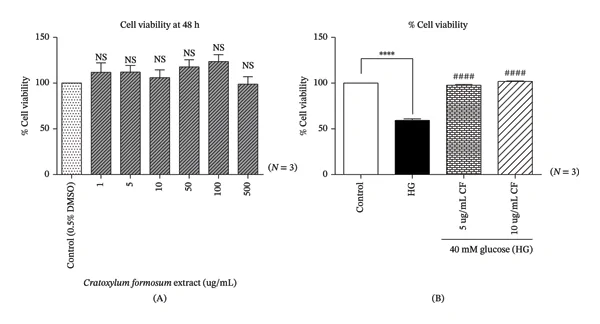
(A) Cytotoxicity test of the effects of CF extract on INS‐1 cells at 48 h. (B) Effects of CF extract against high glucose‐induced toxicity in pancreatic β cells. INS‐1 cells were exposed to 40 mM glucose in the presence or absence of 5 and 10 μg/mL CF extract. After 72 h of treatment, cell viability was determined using the MTT assay. The data are expressed as the mean ± SEM of three independent experiments. Significant differences between groups were determined by one‐way ANOVA with Tukey’s multiple‐comparison test (^∗∗∗∗^ *p* < 0.0001 vs. control; ^####^ *p* < 0.0001 vs. 40 mM glucose).

## 4. Discussion

This study demonstrates the systemic antidiabetic potential of CF leaf extract in an HFD and STZ‐induced type 2 diabetic mouse model. Phytochemical profiling confirmed that the CF extract is rich in phenolics (111.11 mg GAE/g extract), with 5‐CQA (243.74 ± 0.93 mg/g dry extract)‐a major chlorogenic acid isomer‐as the predominant constituent. These findings align with previous reports by Yingngam et al. [8, 9], confirming 5‐CQA as the primary bioactive component of CF extract.

Chlorogenic acids modulate glucose homeostasis by retarding carbohydrate absorption through the inhibition of digestive enzymes [6, 26, 27]. In agreement with Arsakit et al. [13], CF extract displayed dose‐dependent inhibitory activity against α‐amylase (IC50 = 1.14 mg/mL) and α‐glucosidase (IC50 = 1.83 mg/mL). This delayed intestinal carbohydrate digestion provides a primary mechanism accounting for the improved postprandial glycemic control observed during the OGTT.

In addition to enzymatic inhibition, *in vivo* administration of CF extract significantly reduced fasting blood glucose levels (up to 29.31% at 500 mg/kg b.w.) and preserved body weight, skeletal muscle mass, and visceral fat. These outcomes indicate a protective effect against diabetes‐induced metabolic imbalance and muscle wasting. Notably, CF extract restored serum insulin and C‐peptide levels. This functional endocrine recovery correlated with the preservation of islet and pancreatic β‐cell areas. Furthermore, the direct cytoprotective capacity of CF extract under high‐glucose conditions (40 mM glucose) was confirmed *in vitro*, where CF treatment (5–10 μg/mL) significantly restored INS‐1 cell viability. These observations align closely with prior studies on polyphenol‐rich extracts‐including *Eugenia uniflora* [5] and *Cinnamomum zeylanicum* [3, 6]‐and chlorogenic acid [28, 29], which exhibited comparable capabilities in preserving β‐cell viability and endocrine function under diabetic and high‐glucose conditions.

A novel aspect of this study is the effect of CF extract on circulating NPY. NPY is an orexigenic peptide synthesized predominantly in the hypothalamus to regulate appetite and energy balance [30, 31]. Under diabetic conditions, insulin deficiency impairs central NPY suppression, leading to hyperphagia and hyperglycemia [10, 32, 33]. Here, diabetic mice exhibited elevated serum NPY levels, which were significantly reduced toward normal levels following treatment with 500 mg/kg b.w. CF extract. As we know, insulin acts as a negative regulator of NPY expression [32]; this systemic reduction in serum NPY likely represents a secondary consequence of restored endocrine pancreatic β‐cell function and endogenous insulin secretion [34]. Thus, to confirm direct central modulation of NPY requires future quantification of hypothalamic NPY mRNA expression and upstream insulin signaling molecules (e.g., p‐IRS1, *p*‐AKT) via RT‐qPCR and Western blotting.

Unlike acarbose, which acts primarily as a localized intestinal enzyme inhibitor, CF extract displays a broader, multitargeted therapeutic profile. Its antidiabetic efficacy stems from complementary mechanisms: delaying carbohydrate digestion, preserving pancreatic β cell and restoring endocrine‐neuroendocrine balance.

Despite these promising functional outcomes, several limitations should be acknowledged. First, the findings remain largely phenotypic and histomorphometric. Although pancreatic β‐cell preservation suggests attenuated oxidative stress or inhibited apoptosis, key intracellular cascades‐including reactive oxygen species (ROS) levels, lipid peroxidation (MDA), antioxidant enzyme activities (SOD and CAT), and apoptotic regulatory markers (Bcl‐2, Bax, and cleaved caspase‐3)‐were not directly quantified. Likewise, confirming direct central modulation of NPY requires future quantification of hypothalamic NPY mRNA expression and upstream insulin signaling molecules (e.g., p‐IRS1, *p*‐AKT) via RT‐qPCR and Western blotting.

Second, the selection of the 500 mg/kg b.w. dose, while establishing maximal efficacy, presents important limitations regarding translational feasibility. In our study, daily administration of 500 mg/kg b.w. CF extract for 4 weeks in normal mice induced no mortality, which aligns with our *in vitro* findings showing noncytotoxicity in INS‐1 cells up to 500 μg/mL. However, given the high extract load, the lack of full subchronic toxicity assessments—including serum hepatorenal biomarkers (ALT, AST, BUN, and creatinine) and nontarget organ histology—is an acknowledged limitation. Consequently, the 500 mg/kg dose should be interpreted as a proof‐of‐concept for peak bioactivity rather than a directly translatable therapeutic dose. Comprehensive dose‐range optimization and standardized safety evaluations following established OECD guidelines will be imperative before any clinical application can be proposed. Finally, seasonal variations in phytochemical yields, limitations of 2D histomorphometric area analysis, and nonblinded daily dosing (mitigated by blinded biochemical/histological analysis) warrant consideration in future standardized trials.

## 5. Conclusions

The findings of this study suggest the potential value of CF leaf extract in modulating diabetes‐related metabolic dysfunction. In preclinical models, the extract demonstrated antidiabetic effects associated with the inhibition of carbohydrate‐hydrolyzing enzymes, attenuation of hyperglycemia, and favorable outcomes on pancreatic β‐cell function and tissue preservation. Administration of CF leaf extract at 500 mg/kg b.w. exhibited the most pronounced glycemic improvements, accompanied by partial preservation of insulin and C‐peptide levels, alongside suppression of serum NPY. Further research is necessary to identify the specific bioactive constituents, elucidate the underlying molecular mechanisms, confirm safety following chronic administration, and evaluate its translational relevance in clinical settings. Overall, these preliminary findings support CF leaf extract as a candidate for further investigation into the molecular mechanism underlying glycemic control and pancreatic protection.

## Author Contributions

Kanchana Suksri conceptualized and administered the project, performed the animal experiments, and wrote the original draft of the manuscript. Nipaporn Muangchan analyzed the data, prepared the figure, and wrote the original draft of the manuscript. Suwattanee Kooptiwut supervised the project and edited the manuscript. Anchalee Vattarakorn performed the animal experiments. Bancha Yingngam performed the plant collection and extraction. All the authors reviewed the manuscript.

## Funding

This research was supported by the Office of the Permanent Secretary, Ministry of Higher Education, Science, Research and Innovation, Thailand (Research Grant for New Scholars to Kanchana Suksri with Grant No. RGNS 65–124).

## Ethics Statement

All animal experiments were reviewed and approved by the Institutional Animal Care and Use Committee (IACUC), Faculty of Medicine, Siriraj Hospital, Mahidol University (Certificate of Approval No. 017/2023).

## Conflicts of Interest

The authors declare no conflicts of interest.

## Data Availability

The datasets generated and analyzed during this study can be obtained from the corresponding author upon reasonable request.
